# Public health is Indigenous: design and launch of the NW NARCH research academy for American Indian high school students

**DOI:** 10.3389/fpubh.2025.1523998

**Published:** 2025-03-18

**Authors:** Celena Ghost Dog, Amanda Kakuska, Stephanie Craig Rushing, Grazia Cunningham, Allyson Kelley

**Affiliations:** ^1^Northwest Portland Area Indian Health Board, Portland, OR, United States; ^2^Allyson Kelley and Associates PLLC, Sisters, OR, United States

**Keywords:** American Indian, curriculum, pedagogy, public health, evaluation

## Abstract

This article describes the collaborative process of designing the Northwest Native American Research Center for Health (NW NARCH) Research Academy. We describe the NW NARCH partnership-building process with federally recognized tribes in the United States, Washington, Oregon, and Idaho while outlining the goals and steps to indigenize the curriculum. The Research Academy curriculum utilized socioecological models and theoretical frameworks informed by indigenous pedagogies, like the Circle of Courage to further students’ sense of belonging, mastery, independence, and generosity. NW NARCH recruited four sites in 2023 with tribal representation across the Pacific NW. From October 2023 to April 2024, NW NARCH delivered 11 virtual (live) sessions. Our evaluation of the Research Academy included a student-driven evaluation plan, a visual logic model, student and mentor surveys, and Canvas (online learning platform) page views. Preliminary findings and lessons learned from the first cohort of Research Academy students demonstrate that NW NARCH successfully built a public health pathways program for American Indian and Alaska Native students. The Research Academy successfully connected AI/AN high school students to relatable public health role models from a variety of professional pathways, including university professors and Tribal Epidemiology Center staff. Lessons from this first year of the NW NARCH underscore the importance of community- research collaborations and equity-focused programming for underrepresented racial /ethnic groups.

## Introduction

Public health is indigenous. It always has been and always will be. American Indian and Alaska Native (AI/AN) people have identified and utilized core public health strategies since time immemorial—using cultural teachings to protect the health of people, communities, and the environment. These teachings are rooted in personal, holistic, experiential, place-based, and intergenerational learning to elevate upstream interventions for the wellbeing of all (1). However, colonization, marginalization, structural racism, and settler mentalities largely removed positive cultural experiences and indigenous ways of knowing that integrated a holistic understanding of health for AI/AN people.

As a result, AI/AN people are now the most underrepresented racial/ethnic groups in the US health science, public health, and biomedical science workforce (2–4). In 2021, AI/ANs made up less than 1% of the U.S. population and STEM workforce (1). AI/AN students at various levels of education often are not encouraged to pursue science careers (5–11). Less than 1% of students enrolled in U.S. public health degree programs are AI/AN; AI/ANs are the only racial/ethnic group for whom the percentage enrolled and graduated decreased (by 0.3 percentage points) from 0.7% in 1996 to 0.4% in 2016 (12). Fueling this trend, few AI/AN public health faculty and research role models work in university settings to mentor AI/AN students.

Consequently, there is an insufficient number of AI/AN researchers in the “principal investigator” role who guide behavioral, clinical, epidemiologic, and biomedical studies in AI/AN communities. This gap is closely tied to the low number of AI/AN researchers who have adequate training to address AI/AN health disparities (7, 8, 10, 13–16). A key step in addressing the under-representation of AI/AN students in public health research is to nurture interest in the health sciences before college, ensure a successful transition into college, and improve persistence in science-related disciplines (17). Culturally tailored pathways programs can pave the way and increase a student’s sense of social belonging, self-efficacy, scientific identity, and perseverance toward the achievement of academic and career goals (17–22).

Higher educational attainment across a variety of fields has been linked to reduced health disparities (23). Policymakers, tribal leaders, and communities recognize the importance of education and have called for developing innovative programs and pathways to increase the number of AI/AN students enrolled in medical, dental, nursing, public health, behavioral health, and community health programs. Recommendations include developing undergraduate training programs available to students in local settings and tribal colleges, offering student internships and work experience, providing AI/AN students with native role models and mentors, and ensuring holistic wraparound services to support students along the educational pathway (12).

Indigenous public health pathways programs for AI/AN high school students in urban and rural reservation communities are critically needed to increase the representation of AI/AN students and professionals in public health. This paper describes the process of designing and launching an indigenous public health research pathway program for AI/AN high school students at the Northwest Portland Area Indian Health Board (NPAIHB or Board), by the Northwest Native American Research Center for Health (NW NARCH), called the Public Health Research Academy (referred to throughout as Research Academy or Academy). This formative paper describes the process of designing the Research Academy’s goals and theoretical guides, steps taken to indigenize the curriculum centering indigenous ways of knowing, the selection of learning and teaching modalities, the tools designed to evaluate the process in the first year of the project (2022–2023), and preliminary findings and lessons learned from delivering the Research Academy to the first cohort of students (2023–2024).

### About Northwest Portland area Indian health board and NW NARCH

The Northwest Portland Area Indian Health Board (NPAIHB) is a tribal organization representing 43 federally recognized tribes in Washington, Oregon, and Idaho (NW). The mission of the NPAIHB is to “eliminate health disparities and improve the quality of life of American Indians and Alaska Natives by supporting Northwest Tribes in their delivery of culturally appropriate, high-quality health care.” The NPAIHB’s governing board meets quarterly and is comprised of one delegate per member Tribe. Preparing the next generation of public health professionals and researchers has been a long-standing goal of the Board’s Youth Delegate Program, Adolescent Health Team, and NW NARCH’s Advisory Board. All of the NPAIHB’s research and public health promotion programs are guided by community-based participatory research (CBPR) principles. CBPR acknowledges that health programs, campaigns, and interventions can be strengthened by working in close partnership with communities to design and evaluate programs that enhance the health and well-being of their people (24).

### NW NARCH public health research academy goals and components

In July 2022, NPAIHB collaborated with tribal leaders and health officials in the Pacific Northwest (OR, WA, ID) to develop an application for the NW NARCH grant, which was funded by the National Institutes of Health’s National Institute of General Medical Sciences (Award # S06GM145214). The overall goal of NW NARCH Research Academy is to nurture science identity and develop research skills among AI/AN high school students so they can begin to orient their subsequent college and graduate school pathways in the sciences, particularly population health sciences. Building the Research Academy, we sought to recruit a cohort of 12–16 AI/AN students from high schools in the Northwest. Major components of the year-long Research Academy included:

- An in-person 5-day workshop in Portland, Oregon, hosted by the NPAIHB, Portland State University, and Oregon Health Sciences University before the beginning of the academic school year. The agenda focused on research approaches and methods, local campus tours, and exposure to successful AI/AN researchers who work in public health and population science;
- Training and research experiences collaborating with paid mentors located in their local reservation communities;
- Participation in monthly Zoom-based enrichment sessions focused on AI/AN health topics; qualitative, quantitative, and mixed research methods; population sciences; and building student ‘identity’ as future researchers;
- An end-of-year, virtual research showcase where students present their findings and lessons learned to peers, family, mentors, and faculty;
- Students were also encouraged to share their projects with their school, tribal health department, and/or tribal council to reinforce the mindset of giving back to their community.

A 15-member Curriculum Advisory Board met quarterly via Zoom during the first year to provide support and direction to the NW NARCH Team. The meetings helped identify topical experts and guest speakers for the learning sessions, student engagement and recruitment strategies, and provided guidance on program delivery methods, evaluation measures, outreach, and communication strategies.

## Pedagogical frameworks and methods

The Research Academy curriculum utilized socioecological models and theoretical frameworks informed by indigenous pedagogies, like the Circle of Courage (25), to further students’ sense of belonging, mastery, independence, and generosity. Indigenous pedagogies are deeply rooted in cultures and communities; they represent ways of knowing, learning, and being in the world before colonization (26). Consistent with an Indigenous pedagogical approach, the NW NARCH curriculum was designed to be personal, holistic, experiential, place-based, and intergenerational (27). The NW NARCH Research Academy curriculum centered on indigenous teaching methods and epistemologies to expand on dominant Western ideologies, which often exclude the social and spiritual domains of learning and the intergenerational passing of knowledge and wisdom. Teaching methods used throughout the in-person and learning sessions intentionally included non-linear ways of learning and centered around cultural teachings, opening and closing blessings, wellness moments, linkage to Traditional Ways of Knowing (TOK) by framing cultural stories alongside Western public health topics and methods.

Our recruitment strategy centered on recruiting sites/mentors who would advertise the program to their students. As community educators, they have the best pulse on youth interested in public health/research and the ability to commit to a year-long program. We leveraged our Adolescent Health Team network of tribal contacts in OR, WA, and ID to solicit applications. We hosted information webinars, shared recordings in our messaging, and posted videos on our website and social media platforms. Mentors applied for consideration via an online survey. We prioritized sites in OR, WA, and ID.

**Figure 1:**
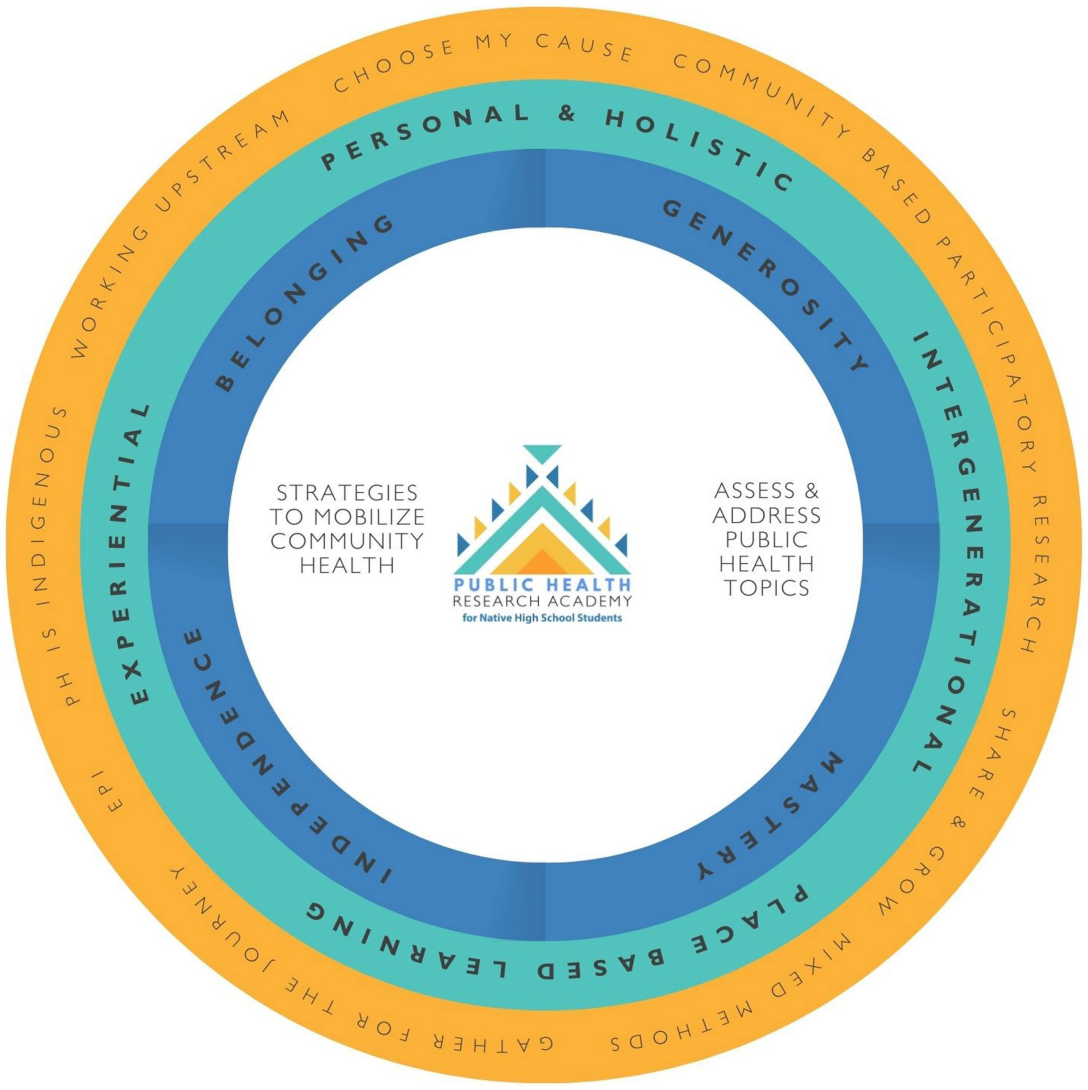
NW NARCH visual model (2023–2024).

**Figure 2:**
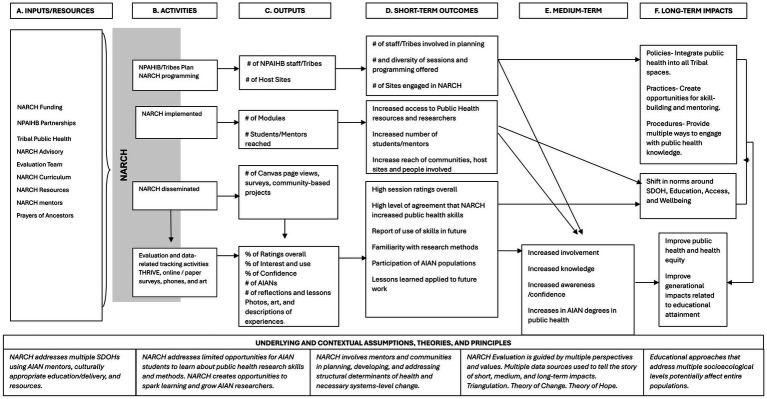
NARCH research academy western logic model (30).

Our evaluation of the NW NARCH Research Academy included a student-driven, pretest-posttest evaluation design. This included a visual logic model, student and mentor surveys, and Canvas page views. Students and their parents/guardians signed a consent form before participating in evaluation activities. No compensation was provided for students or mentors who completed the surveys. The Research Academy Development Team designed a visual logic model to outline the project’s inputs, processes, and goals (28). The visual logic model is similar to a Western logic model where the inputs, activities, resources, outputs, and outcomes follow a linear progression. The map in Figure 1 shows activities, data collection instruments, and inputs on the center line. Figure 2 shows the evaluation process of the NW NARCH Research Academy using a Western Logic Model (28).

The student and mentor surveys were administered online using SurveyMonkey at the beginning of the Research Academy and the end of the year-long experience. The survey questions were designed to explore how the Research Academy nurtured the identity of AI/AN high school students to see themselves as future public health professionals while developing research skills to orient students to academic pathways in the sciences. An external evaluation team used data sources to explore the process and outcome measures for students and mentors involved in the NW NARCH Research Academy (Table 1).

**Table 1:** NW NARCH virtual learning—session titles and content.

Module 1: Indigenous Ways to Health
Handbook Chapters: What is Public Health? What is Research? What does it mean to work upstream? Activity: Prevention Timeline Digital Library: Canvas Help—Video Tutorials, handouts Session Recording
Module 2: Community Based Participatory Research (CBPR)
Handbook Chapters: Community-based Participatory Research Research Method Highlight: Community Needs Assessment & Asset Maps Session Recording
Module 3: Public Health is Indigenous
Handbook Chapters: What are Social Determinants of Health? What’s Community Got to Do with my Health? What’s Law Got to Do with it? Activity: My Indigi Public Health Research Method Highlight: Interviews & Talking Circles Session Recording
Module 4: Indigenized Mixed Methods Research
Handbook Chapters: Indigenized Research Activity: Qualitative Coding Session Recording
Module 5: Winter Social
Activity: Peer Shout-outs Session Recording
Module 6: What Epi Told Me
Handbook Chapters: What is Epidemiology? Epi Principles Activity: Name that Cause! Research Method Highlight: Surveys Session Recording
Module 7: Social Marketing 101
Handbook Chapters: Social Marketing 101, The Four P’s of Marketing, Media Campaigns Activity: Let us Break it Down Research Method Highlight: Brining it all Together! Session Recording
Module 8: Choose & Gather for the Journey
Handbook Chapters: Choose My Cause, Researcher Roles Activity: Resource Map Session Recording
Module 9: Project Workplans
Activity: Workplans Session Recording
Module 10: The Data Say What?
Handbook Chapters: Review Your Data, Analyzing Data, Reflection Time Activity: Site Workplans Session Recording
Module 11: Showcase Prep
Activity: Project Celebration Story Guide Session Recording
Module 12: Share & Grow
Research Project Showcase: Youth presentations Session Recording
Digital Library
Inspirational videos featuring Indigenous researchers and public health role models Skill-building videos featuring Native researchers Videos focused on mentorship from a Native perspective Educational pathway tips and resources

Quantitative survey data were analyzed using descriptive statistics and significance testing of means when appropriate. Specifically, frequencies, means, standard deviations, and paired-sample t-tests were conducted to compare the difference in student responses on identical items in the pre-survey and post-survey. Qualitative data provided in responses to open-ended survey items were analyzed using a general inductive approach framed by indigenous pedagogy (29).

## Learning environment

The in-person and virtual learning sessions were designed to include a broad range of learning styles emphasizing learning by doing. Students were offered a variety of learning tools and formats, including reading materials (Knowledge Seeker News—Handbook Chapters), audio-visual videos (recorded Learning Sessions and Digital Library), individual and small group activities, reflection prompts, and firsthand student-led community-based projects to deepen students’ connection to the learning objectives. All materials are generated by the NW NARCH and not publicly available. The learning environments included live and asynchronous learning sessions, a self-paced Knowledge Seeker Youth Handbook, and a Canvas learning platform [See Supplementary file for Research Academy Curriculum Figure].

The timing, duration, workload, and expectations for participation were aligned with other extra-curricular STEM enrichment programs designed for high school students (See: Pathways Resource Guide^1^).^2^ The workload and expectations for mentors were designed to play to their strengths as tribal educators who connect well with students, are local community members, and are non-researchers who often juggle multiple roles within their community.

A kick-off was offered in June 2023 on the campuses of Portland State University (School of Public Health and the Native American Student and Community Center) and Oregon Health & Science University. The goal of the in-person week was to establish intergenerational relationships among students, mentors, staff, indigenous researchers, research partners, tribal sites and to provide foundational materials about the program. Activities during the week focused on tribal public health topics, potential career pathways, research and evaluation methods, and building financial literacy (e.g., creating budgets, completing W9 forms, etc.; Table 2).

**Table 2:** NW NARCH evaluation data sources (2023–2024).

Data source	Purpose	Number of responses
Youth Baseline Survey	Know who students are, what they are interested in, and previous experiences with research	10
Youth Learning Session Feedback	Document youth perspectives, ratings, usefulness, and takeaways	22
Staff Learning Session Take-aways and Lessons Learned	Document staff perspectives, tweaks for next time, and takeaways	Reflection Log (*n* = 9 sessions)
Mentor Mid-way Feedback Survey	Gather mentor perspectives, satisfaction, and suggestions	4
Youth Year-End Survey	Gather youth impacts and future plans	10
Mentor Year-End Survey	Gather mentor perspectives, satisfaction, and suggestions	4
Canvas Analytics	Document course activity, page views, library views, and student use	1,684 page views
Community-Based Research Projects	Transfer research skills to conduct a youth-led community health research project	6 individual and small group projects

From October 2023 to April 2024, NW NARCH delivered 11 virtual (live) learning sessions to the first cohort of students (*N* = 10). These sessions were hosted and recorded on Zoom. Recordings were posted to the NW NARCH online classroom on Canvas. The twelfth live session was a research showcase dedicated to student presentations of their projects. Virtual learning sessions centered around key public health research topics and indigenous knowledge systems. Sessions featured slideshows, handbook chapters, activities, research methods (pulled from a Data Collection Guide), and offered individual and group support to students and mentors.

Each session lasted 60 min and included a 20-min presentation by an AI/AN subject matter expert who shared practical examples of research methods used while conducting CBPR. Time was allotted at the end of each session for questions and answers and time was provided for the youth to complete the feedback survey. The site mentors attended virtual sessions to facilitate discussions, support learning, and provide technical assistance. Additionally, NARCH facilitators provided 15-min office hours before and after each session to answer questions and build rapport with participants. Frequent updates and reminders were provided to the youth and mentors via email and text message between sessions to offer guidance and answer questions.

Our Canvas digital classroom optimized asynchronous learning for students who could not attend the live sessions. The Academy course on Canvas included discussion boards, digital materials, such as PowerPoint slides, and Knowledge Seeker Youth Handbook materials. We also offered a Digital Library on Canvas that featured inspirational videos with Indigenous researchers, skill-building videos (e.g., data science lessons), and educational resources (e.g., timelines for applying to college), digital literacy tips, and mentorship videos from indigenous public health researchers (Table 3).

**Table 3:** NW NARCH in-person kick-off week—titles and content.

Day 1: Welcome to the Academy!
Meet the Academy Team: Our Journey Together What is Public Health Research? Youth Lead Evaluation
Day 2: Public Health Pathways
Financial Literacy: Blank W9, Blank budget form Panel: Meet Indigenous Public Health Researchers Public Health is Indigenous Explore Story Maps & Photovoice Collection Evening Activity: Cultural Sharing Night
Day 3: Indigenous Ways to Health
What is an EpiCenter? Policy in Public Health Center Us: The State of Native Youth Field Trip: 7 Waters Canoe Family—Working Upstream, Practicing Canoe Culture
Day 4: Public Health is Indigenous
Site Visit: Oregon Health & Science University (OHSU), NW Native American Center of Excellence (NNACoE) & Wy’East Medicine Pathway, OHSU Community Garden Panel: Inspired Social Marketing Campaign Showcase Prep: Public Speaking Tips
Day 5: Project Showcase
Group Project Showcase Presentations

## Results

NW NARCH recruited four sites in 2023 with tribal representation across the Pacific NW. Ten AI/AN students completed the baseline survey; 100% were female, representing five different tribes in Oregon, Washington, and Idaho. Most were entering 11^th^ or 12^th^ grade; one student was entering 10th grade. The first cohort of students participated in the virtual learning component from October 2023 to May 2024 (Table 4).

**Table 4:** NW NARCH research academy sites, locations, student, and mentor representation.

Host site	Location	# of students	# of mentors
Heritage University (mentoring H.S. students)	Toppenish, WA	2	1
Nixya’awii Community School	Pendleton, OR	2	1
Clarkston High School	Clarkston, WA	3	1
Madras High School	Madras, OR	3	1

NW NARCH tracked student engagement with the Academy online classroom materials and Digital Library on Canvas. Canvas analytics shows the number of page views by each student. The total number of Academy classroom page views was 1,586, and the total number of Digital Library page views was 98. Page views were the primary method for tracking engagement, followed by participation in Zoom meetings.

Feedback collected from students and mentors demonstrates overall reflections on the Academy, areas for improvement, and recommendations for the next cohort.

Survey links and QR codes were shared with students after each learning session. A total of 22 surveys were completed by seven students from October 2023 through April 2024. Feedback was provided on seven learning sessions (Choose and Gather, CBPR, Indigenous Ways to Health, Mixed Methods Research, Project Workplans, Public Health is Indigenous, Social Marketing, The Data Say What, and What Epi Told Me). Students rated sessions based on a 10-point scale where 1 is the lowest rating and 10 is the highest; the average rating for all sessions was 9.27 (SD = 0.86, range, 7 to 10). Guest speakers were above average, 85% (*n* = 17) selected, “Above average, invite them back for sure.”

Students rated supplemental learning materials using a Likert-type scale of: *Perfect, Okay, It could use Improvement, N/A.* Responses show high ratings for all of the teaching tools: the Knowledge Seeker News Youth Handbook was rated perfect by 100% (*n* = 20) of students, the Practice in Action Activity and Research Method Highlight were rated perfect by 84% (*n* = 16) of students, and the Reflection Activity was rated perfect by 75% (*n* = 15) of students. Few students rated curriculum supplements as okay, and no students rated them as *needs improvement*. When asked, “How confident are you that you could explain what you learned today to a parent or friend?” 82% (*n* = 18) were very confident, and 18% (*n* = 4) felt they could explain a little. This question is tied to peer-to-peer learning, a value/learning modality we are weaving into the program by bringing back the near peers to help teach the next cohort of students.

Qualitative feedback from open-text responses shows deep appreciation to NW NARCH for the learning sessions and the program while endorsing learning objectives. Themes from qualitative responses include key learnings and appreciation. Students appreciated what they learned about media campaigns, asset mapping, prevention, talking circles, epidemiology, work plans, planning, communication, and public health. One student wrote, “We learned how to do different projects to help keep the community healthy and help us with any negative or positive impacts.” Students learned about public health core concepts throughout the learning sessions, “Public health is not just based on what happened in a clinic; most of your health is determined by other factors such as where you live, work, learn, and play. Some big keywords I took from this session were relationship, reciprocity, and responsibility!” Another student wrote, “This is an amazing program, and I am so grateful for the opportunities it has provided me and the amazing people I have met!” These qualitative responses underscore the importance of NARCH learning objectives to introduce students to population health sciences research using relatable public health topics and challenges.

In addition to the learning session feedback forms, we collected a pre- and post-survey from each student participant to assess outcomes and impacts from the Academy. Interest in public health as a career choice varied at baseline; 50% expressed interest in public health nursing, 25% expressed interest in being a biologist, health educator, public health physical, or social worker, and 25% expressed interest in being an epidemiologist, first responder, and researcher. Their familiarity with public health research methods also varied at baseline; 38% used or practiced surveys, group groups, talking circles, and interviews, and 12% used or practiced community needs and resource assessment. At baseline, no students were familiar with community health projects, photovoice, or StoryMaps. After participating in the Research Academy, 67% reported they had used or practiced surveys, focus groups/talking circles or interviews; 30% reported they had used or practiced a community needs/resource assessment; and 10% reported they had used or practiced a community health project, photovoice, or story maps.

When asked, “How did the Research Academy impact you?” students reported increased connection: 100% felt more connected to their tribe, school, and community; 80% felt more supported; 70% felt more connected to academic resources. Students also reported improvements in knowledge and confidence: 70% reported increased knowledge about public health and research methods, 80% felt more confident, and 70% felt good about where they come from and their future.

When asked, “How will you use the skills you learned at the Research Academy?” 90% reported they will share information they learned in the Academy with their community, 60% will use it to achieve their goals and dreams for the future, 40% believed they will do better in school and academics, and 40% hope to use it to pursue a STEM degree (science, technology, engineering, or math).

Among the student participants, 100% plan to attend college to pursue degrees in public health or medical fields. While participation in the Research Academy increased knowledge and skills in public health, students reported the same likelihood of pursuing a career in public health before and after the program. Before starting the program, students were most interested in biology or environmental health, communications, social marketing, and being a scientist or researcher. At the end of the program, 40% reported they plan to go to college to study public health exclusively, and 40% believe they will use the research methods they learned in their job/career.

We administered a mid- and post-survey to mentors to assess student outcomes and impacts. All four mentors (*n* = 4) provided feedback on the changes they observed in the student participants and what they would like to see in future programs. All four mentors rated the NARCH Research Academy 10/10.

When asked about changes they observed in their student participants, 75% reported that their students were more knowledgeable about public health as a career option at the end of the program, 75% believed their students were more prepared to advocate for themselves and their community, and 25% believed their students had more focus and a better outlook for the future after the Academy. Mentors also expressed enthusiasm for the program in a qualitative, open-ended field at the end of the survey: “I thought this NARCH Program was amazing! It allowed students to research something they were passionate about and sparked more interest in those topics to share with others.”

### Early lessons learned

From this work, there are three lessons learned that NW NARCH will utilize as they plan future cohorts of learning. These lessons are related to asynchronous learning, in-person kick-off meetings, and virtual learning sessions. First, asynchronous learning is essential for reaching students who are busy with sports, classes, and family activities. NW NARCH should continue to offer a variety of learning modalities, including Canvas + Knowledge Seeker Youth Handbook + Recorded sessions as options for student learning. Second, the in-person kick-off meeting was an important start to the partnership between students, NW NARCH, and their mentors. Minor changes to the kick-off week include hosting the community project planning and “firsthand” learning activities to the in-person kick-off week meeting. Finally, virtual learning sessions are essential for students to engage with the NW NARCH Team, but 50% of mentors felt their student participants were overwhelmed and/or overcommitted with competing priorities from school and sports. For the second cohort, NW NARCH will schedule fewer learning sessions and add options for informal check-ins.

### Strengths and limitations

NW NARCH Research Academy is the first ever culturally tailored year-long public health pathway program for AI/AN high school students living in the Pacific NW (Idaho, Oregon, Washington). A strength of the NW NARCH Program is that it builds on the relationships, values, and generational intentions of the NPAIHB, multiple programs, and people. This evaluation reports on the first cohort of NW NARCH students and mentors. All students in the first cohort were female, and this may be due to how students were recruited at various sites by mentors and other programs. It also may be explained by the tendency for females to choose public health careers more than males. As more data are collected from future cohorts, additional information will emerge about how to grow the public health career pathway for AI/AN students.

Initially, the NW NARCH curriculum was to be delivered using synchronous learning methods with live opportunities for speaker and student Q&A engagement. Using page views to track student engagement was the best available metric at the time of this report, future NARCH cohorts will include other metrics of engagement like homework, participation in Zoom meetings, and documentation of engagement in mentoring activities. Due to demanding schedules and previous commitments, many students did not attend the live sessions. This required NW NARCH staff to modify their approach. It also limited some relationship-building benefits of meeting guest speakers and peers during the live Zoom sessions. Completion of the learning session feedback surveys was limited. Only nine of the 12 learning sessions received feedback from students, and 70% completed the post-session feedback forms. Exploring ways to increase student participation in the session feedback surveys could improve our understanding of what they learned from each session.

## Discussion

AI/AN high school students involved in the year-long Research Academy learned that public health is primarily indigenous. They saw this reflected in the topical experts and guest speakers who taught them about public health and the community-based participatory research methods they used. Following an indigenous pedagogy, students reflected on core public health principles and practiced public health research methodologies—learning their practical use in tribal settings. The Academy successfully connected AI/AN high school students to relatable public health role models from a variety of professional pathways, including University professors and Tribal EpiCenter staff. The final student showcase highlighted a number of lessons gained by students while conducting their own student-led CBPR project, including the importance of getting support from trusted Elders and community mentors along the way.

Importantly, the Research Academy was designed to meet high school students where they are, welcoming students with a wide range of competencies into the program. Many students in the first cohort gravitated to one or more learning modalities. Some visibly excelled in the Canvas learning environment, others attended and participated in the live learning sessions via Zoom, others watched the recorded sessions (individually or with their small groups), and many referenced the Knowledge Seeker Youth Handbook during and after the learning sessions. The importance of offering various learning modalities and environments for students to connect with was a clear takeaway from the Year 1 launch. Students reported improvements in both public health knowledge and academic confidence. All students plan to pursue public health or medical degrees in college, demonstrating a positive outcome of the Research Academy on student confidence and aspirations in the field. Mentors also rated their participation in the Research Academy highly and expressed an interest in participating in future years.

The NPAIHB remains committed to inspiring and preparing the next generation of AI/AN public health heroes and supporting capacity-building efforts for students and mentors across the academic pathway. The Healthy Native Youth website is one such resource designed for tribal health educators and teachers. The lesson plans housed on the site embrace cultural teachings and promote positive student development. Several pathway-related resources have already been added to the website:

- Public Health 101 and Internship Guides: https://www.healthynativeyouth.org/stand-alone-lessons/#pathways
- Tribal Public Health Pathways Resource Guide: https://www.healthynativeyouth.org/resource/tribal-public-health-pathways-resource-guide/
- Inspire Text Messaging Campaign: https://www.healthynativeyouth.org/resource/text-inspire-to-94449/
- Inspire YouTube Playlist: https://www.youtube.com/playlist?list=PLvLfi7yZ2zQFB1f4kGjiG9ipsiFAvi5kx

Additionally, the Public Health Research Academy curricula lesson plans will be re-formatted for use by schools and tribal educators next year and made available on the Healthy Native Youth website.

### Implications

Students in the NW NARCH Public Health Research Academy learned about critical public health issues relevant to the NW Tribes. They gained firsthand experience using indigenous research methods to carry out a community-based project. While doing so, the program increased student’s connections and confidence and enriched their academic pathways and career aspirations. In the future, NARCH will work to diversify student genders, document student engagement more fully, and follow up with students to determine the overall impact of participation in NARCH on their pursuit of public health degree programs. This Research Academy will help pave the way for future generations of Indigenous health researchers, where high school students connect with mentors, meet professional role models, grow their research capacities, and improve health outcomes for all.

## Data Availability

The raw data supporting the conclusions of this article will be made available by the authors, without undue reservation.

## Footnotes

^1^ https://www.wernative.org/my-pathway

^2^ https://www.healthynativeyouth.org/resource/tribal-public-health-pathways-resource-guide/
