#  Erratum: Public health is Indigenous: design and launch of the NW NARCH research academy for American Indian high school students

**DOI:** 10.3389/fpubh.2025.1611344

**Published:** 2025-05-01

**Authors:** Frontiers Production Office

**Affiliations:** Frontiers Media SA, Lausanne, Switzerland

**Keywords:** American Indian, curriculum, pedagogy, public health, evaluation

Due to a production error, there was a mistake in the title as “indigenous” was not capitalized. The correct title is “Public health is Indigenous: design and launch of the NW NARCH research academy for American Indian high school students.”

Furthermore, an author's surname was incorrectly listed as only “Dog”. The correct full surname is “Ghost Dog”. In the Acknowledgments, the title “Dr.” was not inserted in front of the names Tom Becker and Cliff Poodry. Their correct naming is Dr. Tom Becker and Dr. Cliff Poodry.

Due to a production error, the reference to “Kelley A. Public health evaluation and the social determinants of health. Abingdon, Oxon: Routledge (2020)” was omitted. The reference has now been added to the reference list and to the end of the caption of Figure 2. There was a mistake in Figure 2 as published. The “Kelley, 2020” was removed from the Figure image. The corrected Figure 2 and caption appear below.

**Figure 2:**
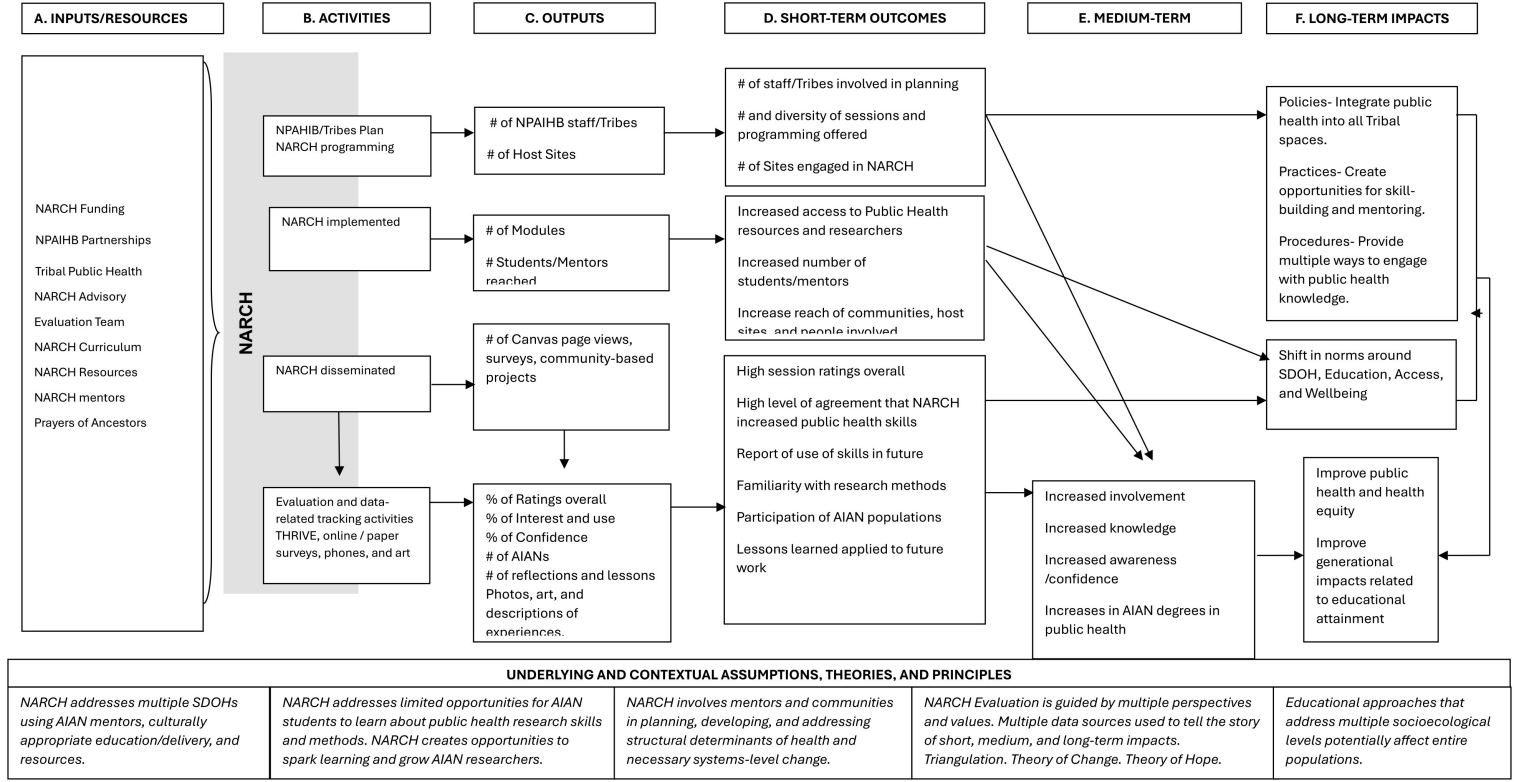
NARCH research academy western logic model (30).

Finally, the Supplementary material [Supplementary Image 1] was omitted. The file has now been published.

The publisher apologizes for these mistakes.

The original article has been updated.

