# Supplementary material for: Public health is Indigenous: design and launch of the NW NARCH research academy for American Indian high school students
Source: Front Public Health. 2025 Mar 18;13:1523998. doi: 10.3389/fpubh.2025.1523998 (PMC11962262; doi:10.3389/fpubh.2025.1523998)
Supplement: Supplementary file 1 [file Table_1.DOCX]

Online Links and Videos

H.S. NARCH [Public Health Research Academy](https://www.npaihb.org/public-health-research-academy/)

The Academy is featured this month on We R Native’s YouTube channel: [https://www.youtube.com/watch](https://www.youtube.com/watch?v=sKr8s540JAI&list=PLvLfi7yZ2zQFB1f4kGjiG9ipsiFAvi5kx)

Our youth-driven Evaluation Process was also recently featured in the [Indigenous Peoples in Evaluation eNewsletter](https://higherlogicdownload.s3.amazonaws.com/EVAL/9bfc598e-108e-464e-a54c-f905669e2811/UploadedImages/October_23_IPE_Tig_Newsletter_Template__1_.pdf)

Day 1 video on the NARCH track: <https://www.youtube.com/watch?v=7wdve7JhkYg>
