# Supplementary figures and images for: Public health is Indigenous: design and launch of the NW NARCH research academy for American Indian high school students
Source: Front Public Health. 2025 Mar 18;13:1523998. doi: 10.3389/fpubh.2025.1523998 (PMC11962262; doi:10.3389/fpubh.2025.1523998)

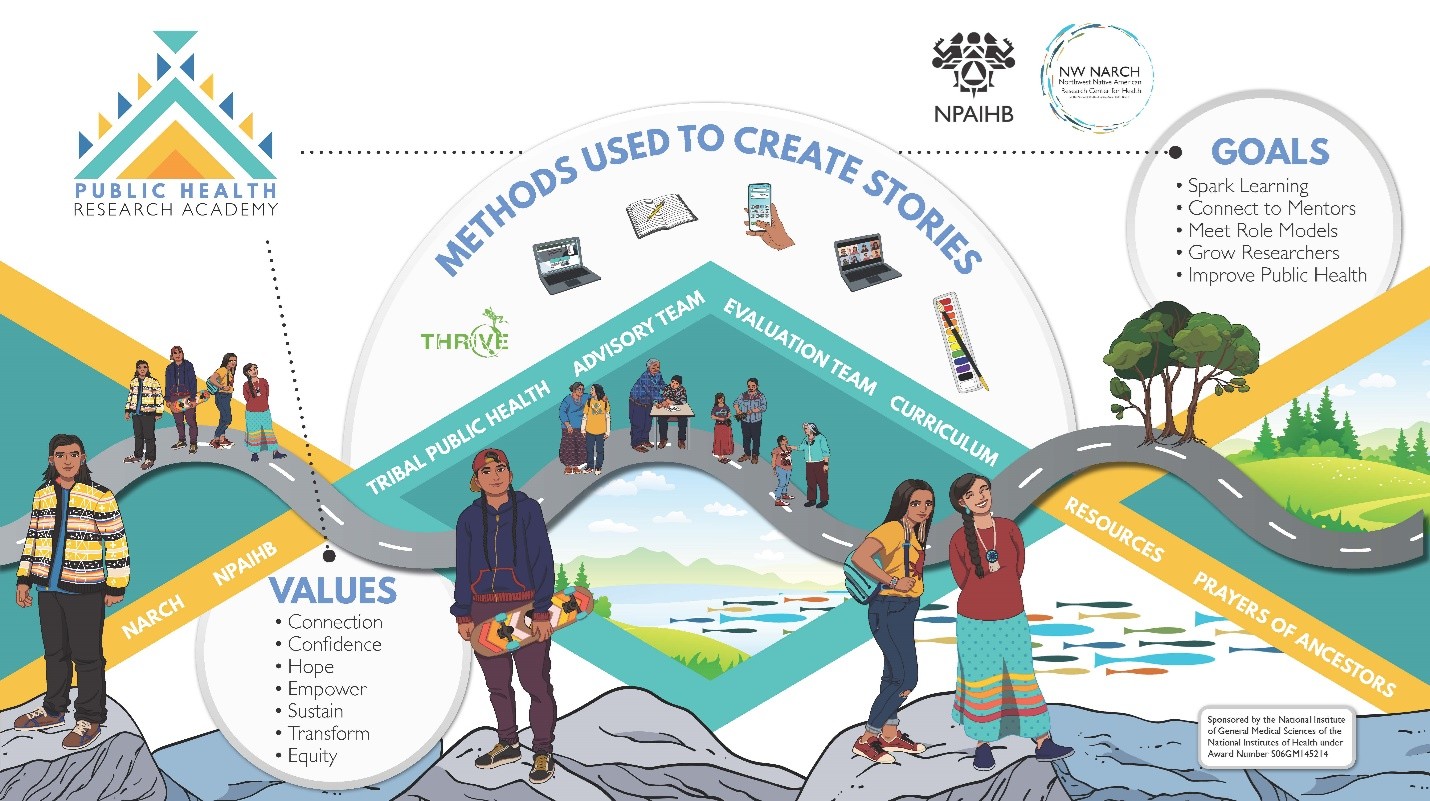

Supplement: Supplementary file 2 [file Image_1.JPEG]
